# Linking serum leptin and TSH among metabolic syndrome patients with and without hypothyroidism

**DOI:** 10.6026/973206300210006

**Published:** 2025-01-31

**Authors:** Barla Krishna, Priya K Dhas, Veluri Ganesh

**Affiliations:** 1 Department of Biochemistry, Vinayaka Mission's Kirupananda Variyar Medical College and Hospitals, Vinayaka Mission's Research Foundation (Deemed to the University), Salem, Tamil Nadu, India; 2 Department of Biochemistry, PES University Institute of Medical Sciences and Research, PES University, Bangalore - 560100, Karnataka, India

**Keywords:** Hypothyroidism, leptin, metabolic syndrome, thyroid stimulating hormone(TSH)

## Abstract

The comparison of thyroid profile and serum leptin levels among patients with metabolic syndrome who did not have hypothyroidism is of interest. Hence, we recruited 40 healthy controls and 80 patients with metabolic syndrome where 40 patients had hypothyroidism and 40 patients are without hypothyroidism. Blood sugar levels, thyroid profiles, lipid profiles and serum leptin levels were measured and compared between the metabolic syndrome and control groups. The serum leptin levels of patients with hypothyroidism were considerably higher (P=0.0001**) when compared to metabolic syndrome patients without hypothyroidism and controls. Furthermore, there was a substantial positive correlation between leptin levels and thyroid stimulating hormone (TSH). Results show that serum leptin and TSH are significantly correlated among metabolic syndrome patients with and without hypothyroidism. Thus, monitoring of leptin levels among metabolic syndrome patients with and without hypothyroidism is relevant.

## Background:

Metabolic syndrome is a chronic metabolic disease caused by abnormality in the carbohydrate, lipid and increased blood pressure. A significant risk factor for both cardiovascular disease (CVD) and type 2 diabetes mellitus is metabolic syndrome [1- 2]. In particular, a threefold rise in metabolic syndrome prevalence is linked to a fivefold increase in the chance of diabetes mellitus, a twofold increase in the risk of dying from cardiovascular disease and a 150% increase in overall mortality [3-4]. Early detection of metabolic syndrome and the ensuing intervention measures may help lower the occurrence of these related diseases because metabolic syndrome is linked to an increased risk for diabetes mellitus and cardiovascular disease [5- 6]. Adipose tissue is the primary site of production for the protein hormone leptin, which controls its mass by influencing food intake and energy metabolism. It has its own receptors spread throughout the body and the methods by which it functions are starting to be understood [7-8]. It also has a role in other physiological processes, such as reproduction. Adiposity has a significant impact on leptin levels because the volume of body adipose tissue is related to the quantity of leptin released by white adipose tissue [9- 10]. Leptin production and expression are also influenced by a number of other factors, including as sleep, body temperature, gender, circadian rhythm, fast or excessive meal consumption and other hormones like insulin, growth hormone, glucocorticoids, testosterone and thyroid hormone [11-12]. One characteristic of obesity and abdominal adiposity, which is a risk factor for metabolic syndrome, is elevated serum leptin levels. Despite not being one of the diagnostic criteria for metabolic syndrome, high blood leptin is elevated in individuals with metabolic syndrome [13]. Elevated serum levels of leptin are frequently found in human obesity due to the hormone's degree of influence on hunger, energy consumption, adipose production and insulin function [14]. Serum leptin levels increase with body mass index (BMI) or waist circumference. Other cardiometabolic risk factors, including type-2 diabetes, insulin resistance and hypertension, have been repeatedly linked to high levels of circulating leptin. Nevertheless, not much research has examined its connection to metabolic syndrome [15]. Therefore, it is of interest to evaluate the predictive usefulness of leptin levels for identifying individuals with metabolic syndrome as well as the relationship between leptin and metabolic syndrome and cardiovascular disease risk.

## Materials and Methods:

The current analytical cross-sectional study was conducted in the biochemistry and medicine department of Gayatri Vidya Parishad Medical College in Visakhapatnam Andhra Pradesh, India. According to the International Diabetes Federation (IDF), 80 metabolic patients were diagnosed [16]. These patients were then further divided into two groups based on their thyroid status: Group 2: Metabolic syndrome without hypothyroidism (n = 40) and Group 3 with hypothyroidism (n = 40). Furthermore, we added forty (40) healthy controls that were matched for age, gender and body mass index (BMI); they were referred to as Group 1. The institutional ethics committee (IEC, Ref No: GVPIHCMT/IEC/20201012/02) approved the study's conduct and the study participants were chosen after signing an informed consent form.

## Criteria of the study:

## Inclusion criteria:

The study participants should be between the ages of 30 and 70 and diagnosed with hypertension, elevated fasting blood glucose and a lipid profile in accordance with international diabetes federation criteria. Increased thyroid stimulating hormone (>8 µIU/mL) was used to diagnose hypothyroidism in the healthy controls who were free of disease.

## Exclusion criteria:

The study excludes participants who are pregnant or nursing, have acute or chronic infectious disorders, liver or kidney disease, heart disease, hyperthyroidism, or urinary tract infections, or who are unwilling to participate.

## Blood sample collection:

Each study participant had five (5) millilitres of blood extracted overnight, which were divided into three (3) plain tubes, two (2) Ethylene Diamine Tetraacetic Acid tubes and one (1) fluoride tube. The serum and plasma samples were separated by centrifugation and then transferred into aliquots with the proper labels. Until they are examined, the aliquots are stored at -50°C.

Standard laboratory procedures were used to determine the results of routine laboratory tests, such as blood sugar levels and lipid profiles, including total cholesterol, triglycerides and high-density lipoprotein. Using the Chem Ultra-Euro Fully automatic analyzer, Mindray CL-1200i and Immuno Assay Automatic Analyser, the Enzyme Linked Immuno sorbent Assay was used to measure total T3, T4 and Thyroid Stimulating Hormone (TSH) and leptin. The immunoturbidimetric method was used to estimate the glycated haemoglobin (HbA1c).

## Statistical analysis:

The distribution was reported as mean ± standard deviation (SD) and examined using the Kolmogorov Smirnov Test was use of analysis of variance (ANOVA) to compare the variables between the groups. Pearson's correlation analysis was used to determine the relationship between the study participants' serum levels of leptin and other factors. A P value of less than 0.05 was deemed statistically significant. The statistical package of social sciences (SPSS) and Microsoft Office Excel were used for all statistical analysis.

## Results:

The study subjects' anthropometric, demographic and biochemical characteristics are shown in Table 1. The study participants' blood pressure, body mass index and age all differed significantly (P<0.05). When compared to controls, metabolic syndrome with and without hypothyroidism was associated with significantly higher levels of fasting and postprandial blood sugar, triglycerides total cholesterol, very low-density lipoproteins (VLDL) and low-density lipoproteins (LDL) (P<0.05). Furthermore, we found that individuals with metabolic syndrome, both with and without hypothyroidism, had significantly lower high-density lipoprotein levels than controls (P<0.05). Comparing metabolic syndrome with hypothyroidism to controls and metabolic syndrome without hypothyroidism, there was a substantial rise in thyroid stimulating hormone levels (P=0.0001**). The study subjects' experimental characteristics are shown in Table 2. The serum insulin, HOMA-IR levels significantly higher in metabolic syndrome patients with hypothyroidism when compared to without hypothyroidism and controls. There was a significantly increased level of malondialdehyde in metabolic syndrome patients with hypothyroidism when compared to without hypothyroidism and controls. Additionally, the FRAP levels are significantly decreased in both metabolic syndrome patients with and without hypothyroidism when compared to controls. Furthermore, we also observed there was a significantly elevated level of serum leptin in metabolic syndrome patients with and without hypothyroidism and controls. The Table 3 shows the correlation of serum leptin with other parameters of the study. The serum leptin was significant positively correlated with age, body mass index SBP, DBP, FBS, PPBS, triglycerides total cholesterol, very low-density lipoproteins, LOW-DENSITY LIPOPROTEINS thyroid stimulating hormone and malondialdehyde (MDA) and negatively correlated with high-density lipoprotein and FRAP. The P values are less than 0.0001** & 0.014*.

## Discussion:

In this study, patients with metabolic syndrome who had hypothyroidism and those who did not had significantly higher levels of body mass index SBP, DBP, FBS, PPBS; triglycerides total cholesterol, very low-density lipoproteins, low-density lipoproteins malondialdehyde, insulin and HOMA-IR than controls (Figure 2 - see PDF). Comparing metabolic syndrome with and without hypothyroidism to healthy controls, new research also revealed markedly higher blood sugar, dyslipidaemia and hypertension levels. Further more, we found that, in comparison to patients with metabolic syndrome without hypothyroidism and controls, patients with hypothyroidism had considerably higher levels of thyroid stimulating hormone and a positive correlation with blood sugar, dyslipidaemia and hypertension [17].

Significantly higher serum thyroid stimulating hormone levels have also been linked to metabolic syndrome in earlier research and these findings have been linked to changes in lipid and carbohydrate metabolism, which can result in obesity and obesity-related conditions like Type 2 Diabetes Mellitus (T2DM) and Cardiovascular Diseases (CVD) in both metabolic syndrome patients and controls with and without hypothyroidism [18, 19-20] (Figure 3 - see PDF). Patients with metabolic syndrome were more likely to develop cardiovascular illnesses and biomarkers are needed to detect problems in metabolic syndrome Recent research on serum leptin suggests that it may be utilized as a marker to detect problems in people with metabolic syndrome including as T2DM and cardiovascular disease [21]. The leptin is mostly found in adipose tissue in amounts proportionate to its bulk. It is now understood to be a significant regulator of body weight, triggering different physiological processes based on the energy balance of the body [22]. Serum leptin levels serve as sensors of the energy balance, informing the hypothalamus about the energy stored in adipose tissue. While low levels, which indicate weight loss, stimulate hunger and decrease energy expenditure, high levels cause appetite to decrease and energy expenditure to increase [23]. These processes are disrupted in obese people numerous hormones and neurotransmitters are involved in the physiological processes that underlie leptin production, secretion, receptor binding and its regulation of hunger and energy expenditure, though, as elevated leptin levels do not produce these results [24]. These processes are intricate and poorly understood.

Serum leptin levels were linked to both cardiovascular risk and metabolic syndrome in the current investigation. In particular, compared to individuals with lower leptin levels, those with greater levels exhibited higher metabolic risk factors [25]. The present study also examined the serum leptin levels in metabolic syndrome patients with and without hypothyroidism and controls. We found that, in comparison to healthy controls, the serum leptin levels in metabolic syndrome patients with and without hypothyroidism were significantly higher and positively connected with blood sugar, lipid profile and hypertension (Figure 4 - see PDF). White adipose tissue secretes it and because of its neuroendocrine properties, it helps regulate metabolism. According to certain research, the levels of serum leptin in the bloodstream are primarily responsible for fatty acid metabolism in several tissues, including the brain and are also involved in energy balance, insulin secretion and activation [26- 27]. According to recent research, excessive blood sugar levels, in appropriate insulin secretion and activation and elevated serum leptin levels all contribute to the generation of serum thyroid stimulating hormone [28]. Together, the amounts of thyroid stimulating hormone and leptin in the bloodstream stimulate the enzymes that produce blood sugar, leading to hyperglycemia and dyslipidaemia [29]. Among metabolic syndrome patients with and without hypothyroidism, the current investigation discovered a significant increase in serum leptin levels that were positively connected with blood pressure, blood sugar, lipid profile and thyroid stimulating hormone (Figure 1 - see PDF). Significantly higher serum leptin levels regulate thyroid releasing hormone (TRH) levels, which in turn increases anterior pituitary gland thyroid stimulating hormone production, according to advanced research. According to research findings, leptin may serve as a potential biomarker due to the notable production of leptin levels in patients with metabolic syndrome and hypothyroidism.

## Conclusion:

Results show that serum leptin and thyroid stimulating hormone are significantly correlated in patients with metabolic syndrome irrespective of with and without hypothyroidism. Thus, monitoring of leptin levels among individuals having metabolic syndrome with and without hypothyroidism is relevant.

## Figures and Tables

**Table 1:** Comparison of biochemical parameters among study groups by using ANOVA

**Parameter**	**Group 1**			**Group 2**			**Group 3**			**P-Value**
Age (Years)	20.94	±	2.95	47	±	10.78	45.63	±	4.23	0.0001**
BMI (kg/m^2^)	20.94	±	2.95	31.8	±	4.85	42.97	±	7.94	0.0001**
SBP	124.38	±	5.25	151.58	±	4.31	165.1	±	5.31	0.0001**
DBP	75.45	±	3.3	95.15	±	2.71	89.33	±	9.71	0.0001**
FBS (mg/dL)	83.93	±	6.46	137.1	±	19.37	144.93	±	36.7	0.0001**
PPBS (mg/dL)	133.55	±	5.98	159.33	±	13.06	182.73	±	30.82	0.0001**
HbA1c (%)	4.6	±	0.44	7.25	±	1.1	8.68	±	0.43	0.0001**
Total Cholesterol (mg/dL)	149.75	±	12.42	297.9	±	41.59	317.25	±	32.99	0.0001**
Triacylglycerides (mg/dL)	115.15	±	12.28	301.78	±	27.04	304.23	±	40.46	0.0001**
HDL (mg/dL)	57.28	±	4.54	29.1	±	2.98	28.55	±	2.75	0.0001**
VLDL (mg/dL)	23.03	±	2.46	60.36	±	5.41	60.85	±	8.09	0.0001**
LDL (mg/dL)	69.45	±	12.74	208.45	±	41.81	227.86	±	33.31	0.0001**
TSH (µIU/mL)	2.49	±	1.21	2.19	±	1.28	11.1	±	3.42	0.0001**

**Table 2:** Comparison of experimental parameters among study groups by using ANOVA

**PARAMETER**	**GROUP 1**			**GROUP 2**			**GROUP 3**			**P-Value**
Insulin	9.79	±	1.76	21.15	±	1.85	30.86	±	5.11	0.0001**
HOMA IR	2.02	±	0.34	7.15	±	1.15	11.03	±	3.26	0.0001**
MDA (nmol/mL)	5.34	±	1.23	19.76	±	3.91	32.46	±	4.19	0.0001**
FRAP (mmol/l)	2.66	±	0.8	2.32	±	0.32	2.32	±	0.3	0.005
Leptin (ng/mL)	4.05	±	2.12	11.55	±	3.15	25.18	±	5.46	0.0001**

**Table 3:** Correlation of serum leptin with other parameters of the study

**Parameter**	**R-Value**	**P-Value**
BMI (kg/m^2^)	0.791	0.0001**
SBP	0.836	0.0001**
DBP	0.42	0.0001**
FBS (mg/dL)	0.487	0.0001**
PPBS (mg/dL)	0.635	0.0001**
HbA1c (%)	0.794	0.0001**
Insulin	0.842	0.0001**
HOMA IR	0.732	0.0001**
Total Cholesterol (mg/dL)	0.705	0.0001**
Triacylglycerides (mg/dL)	0.676	0.0001**
HDL (mg/dL)	-0.681	0.0001**
VLDL (mg/dL)	0.676	0.0001**
LDL (mg/dL)	0.699	0.0001**
TSH (µIU/mL)	0.749	0.0001**
MDA (nmol/mL)	0.842	0.0001**
FRAP (mmol/l)	-0.225	0.014*
